# Beyond Intracellular Accumulation of Polyhydroxyalkanoates: Chiral Hydroxyalkanoic Acids and Polymer Secretion

**DOI:** 10.3389/fbioe.2020.00248

**Published:** 2020-04-03

**Authors:** Luz Yañez, Raúl Conejeros, Alberto Vergara-Fernández, Felipe Scott

**Affiliations:** ^1^Green Technology Research Group, Facultad de Ingeniería y Ciencias Aplicadas, Universidad de los Andes, Santiago, Chile; ^2^Escuela de Ingeniería Bioquímica, Pontificia Universidad Católica de Valparaíso, Valparaíso, Chile

**Keywords:** 3-hydroxyalkanaoic acids, polyhydroxyalkanoates, chiral compounds, biosynthesis, metabolic engineering

## Abstract

Polyhydroxyalkanoates (PHAs) are ubiquitous prokaryotic storage compounds of carbon and energy, acting as sinks for reducing power during periods of surplus of carbon source relative to other nutrients. With close to 150 different hydroxyalkanoate monomers identified, the structure and properties of these polyesters can be adjusted to serve applications ranging from food packaging to biomedical uses. Despite its versatility and the intensive research in the area over the last three decades, the market share of PHAs is still low. While considerable rich literature has accumulated concerning biochemical, physiological, and genetic aspects of PHAs intracellular accumulation, the costs of substrates and processing costs, including the extraction of the polymer accumulated in intracellular granules, still hampers a more widespread use of this family of polymers. This review presents a comprehensive survey and critical analysis of the process engineering and metabolic engineering strategies reported in literature aimed at the production of chiral (*R*)-hydroxycarboxylic acids (RHAs), either from the accumulated polymer or by bypassing the accumulation of PHAs using metabolically engineered bacteria, and the strategies developed to recover the accumulated polymer without using conventional downstream separations processes. Each of these topics, that have received less attention compared to PHAs accumulation, could potentially improve the economy of PHAs production and use. (*R*)-hydroxycarboxylic acids can be used as chiral precursors, thanks to its easily modifiable functional groups, and can be either produced *de-novo* or be obtained from recycled PHA products. On the other hand, efficient mechanisms of PHAs release from bacterial cells, including controlled cell lysis and PHA excretion, could reduce downstream costs and simplify the polymer recovery process.

## Introduction

Synthetic plastics produced from petroleum-derived monomers, such as ethylene, propylene, styrene, and polyethylene terephthalate, are non-biodegradable polymers that undergo slow fragmentation to micron-size particles (Kubowicz and Booth, 2017). Nearly 60% of all plastics produced between 1950 and 2015, equivalent to 4,900 metric tons, can be found in landfills or in the natural environment. Another 800 metric tons were incinerated and 600 MT recycled. Only 10% of the recycled plastics have been recycled more than once (Geyer et al., 2017).

The environmental burden imposed by the production of petroleum-derived polymers can be ascribed to its persistence in the environment and its impact on climate change. On the former, evidence suggests a complex toxicology of microplastics on marine life and in the food chain (Worm et al., 2017). Regarding the latter, each stage in the current plastic lifecycle generates greenhouse gases (GHGs): fossil fuel extraction and transport, monomer production, plastic refining and manufacturing, and plastic waste management. Estimations indicate that if plastic production grows at the current rate, by 2050 the cumulative emissions from plastic use could account for over 56 gigatons of carbon. This is equivalent to 10–13% of the remaining carbon budget (Hamilton et al., 2019).

The search for biodegradable polymers with a reduced emission of GHGs has led to the development of microbial-based processes using natural or genetically engineered microorganisms capable of producing polymers (e.g., polylactic acid and polyhydroxyalkanoates) or monomers (lactic acid, succinic acid, caproic acid, and hydroxyalkanoic acids) with the potential of replacing the current petroleum-based methods (Tsuge et al., 2016).

Renewable bio-based and biodegradable polymers are expected to play a key role in curbing the impacts of plastic production and use in the near future (Zheng and Suh, 2019). One of the most studied biopolymers is poly(R-3-hydroxybutyrate), PHB, an intracellular polyester accumulated in many bacteria. PHB is a member of a large family of renewable and biodegradable bio-polyesters collectively known as polyhydroxyalkanoates (PHAs). They are built by (*R*)-3-hydroxy fatty acid monomers varying from 3 to 5 carbon atoms (short-chain-length PHAs, scl-PHA) and from 6 to 14 carbons (medium-chain-length PHAs, mcl-PHA). Although as many as 150 different congeners of PHA are known with different monomers (Steinbüchel and Valentin, 1995), PHB is the most extensively studied and excellent reviews have been published regarding strategies to improve its production (Peña et al., 2014) and its process technology (Alves et al., 2017).

PHB has been studied for decades as a source of a renewable polymer for the substitution of fossil-derived plastics (Chen and Patel, 2012). However, its use as a substitute for thermoplastics has been hampered by high production costs, dominated by the cost of substrates for the fermentation stage and the costs of downstream extraction and purification. The costs of supplying the required carbon and energy source for microbial growth and PHB production are estimated to be between 30 and 50% of the product cost if sugars are used (Choi and Lee, 1999; Levett et al., 2016). On the other hand, the downstream processes rely on hypochlorite (Heinrich et al., 2012) and solvents (Jiang et al., 2018) for the extraction and precipitation of the polymers. Solvent recovery further adds to processing costs and energy use.

Finally, the large variety of monomers that can be incorporated into PHAs makes PHB only one member of a large family of polymers with different physical properties. Costs are further increased by the precursors required for the production of customized thermoplastics, such as poly(3-hydroxybutyrate-*co*-3-hydroxyvalerate) were valerate is used to change the polymer composition (Choi and Lee, 1999).

This review focuses on a simple but powerful concept, multiple carbon substrates can be funneled to poly(hydroxyalkanoates) and its enantiomerically pure monomers, 3-hydroxyalkanoic acids (3HAs). These monomers are a family of related compounds having at least two functional groups (a hydroxy group and a carboxyl group) that are amenable for chemical modification, they can play a role as building blocks for the synthesis of a large number of products, including diols, polyesters, and fine chemicals. These monomers can be produced either by *in-vivo* depolymerization of the accumulated PHAs, by fermentation for the direct production of 3HAs into the culture media and by conversion of purified PHAs. The advantage of the first two alternatives is that production does not require the extraction of the intracellular PHAs.

Here, we first introduce the potential uses of polyhydroxyalkanoates and its monomers (section Potential Uses of Polyhydroxyalkanoates and Its Monomers) followed by the substrates used for its production (section Substrates for the Production of Polyhydroxyalkanoates and Its Monomers). Section Accumulation and Mobilization of PHAs deals with the pathways and mechanisms of PHAs accumulation and mobilization in bacteria, with an emphasis on PHB. In section Production of R-3-Hydroxyalkanoic Acids From PHAs and Alternative Substrates and Microorganisms for 3HA Production, different strategies for the production of 3HAs are discussed, including *in-vivo* hydrolysis of PHAs and genetic modifications aimed at the direct production of these acids without a polymer accumulation step. Finally, section Secretion of PHAs discusses the research on alternative methods for PHAs liberation and recovery from bacteria to supply substrates for 3-hydroxy acids production.

## Potential Uses of Polyhydroxyalkanoates and its Monomers

Polyhydroxyalkanoates (PHAs) have been extensively investigated to identify possible applications. Homopolymers, random copolymers, and block copolymers can be produced depending on the structure of the polymer chain, this is dictated by the species of bacteria and the substrate used for the accumulation of PHA (Braunegg et al., 2004). The diversity of applications is wide, including production of biodegradable plastics that are environmentally friendly for use in packaging (Koller, 2014; Khosravi-Darani, 2015), fibers (Dietrich et al., 2016), biodegradable and biocompatible implants (Misra et al., 2006), drugs and fine chemical (Rathbone et al., 2010), and biofuels (Zhang et al., 2009).

PHAs have been traditionally used in the packaging of a series of products as shampoo bottles, shopping bags, containers and paper coatings, utensils, carpets, compostable bags, and thermoformed articles (Bugnicourt et al., 2014).

As biomedical materials, PHAs have been used in suture materials and repair patches, meniscus restoration devices, cardiovascular patches, orthopedic pins, and cartilage regeneration aids, among others (Volova et al., 2003; Wang et al., 2005). Many of these uses are related to the customizable composition and properties of PHAs, which allow them to have favorable mechanical properties, biocompatibility, and to degrade in reasonable times under specific physiological conditions (Misra et al., 2006; Hazer et al., 2012). In particular, mcl-PHAs have potential applications in coatings and in medical temporary implants such as scaffoldings for the regeneration of arteries and nerve axons (Witholt and Kessler, 1999). On the other hand, the use of these polymers has been studied in controlled drug delivery (Shah et al., 2010). The kinetics of drug release can be engineered by altering the degradation rate of the PHA matrix coating. In this regard, mcl-PHA have been used as drug carriers since its low fusion point and low crystallinity makes them suitable for controlled drug release (Ueda and Tabata, 2003).

Finally, PHA derived compounds can be used as biofuels after the esterification of PHB and mcl-PHAs with methanol for its conversion to hydroxyalkanoate methyl esters (Zhang et al., 2009). These hydroxyalkanoate methyl esters can be mixed with gasoline and diesel in ratios of 10 to 30%. In particular, (*R*)-3-hydroxy-methyl-butyrate was reported to have similar or improved properties as a fuel additive (oxygen content, dynamic viscosity, flash point, and boiling point) compared to ethanol (Wang et al., 2010). Using PHA derivatives as biofuels can be viewed as a promising application since mixtures of PHA can be used without a costly separation step (Gao et al., 2011).

**Figure 1:**
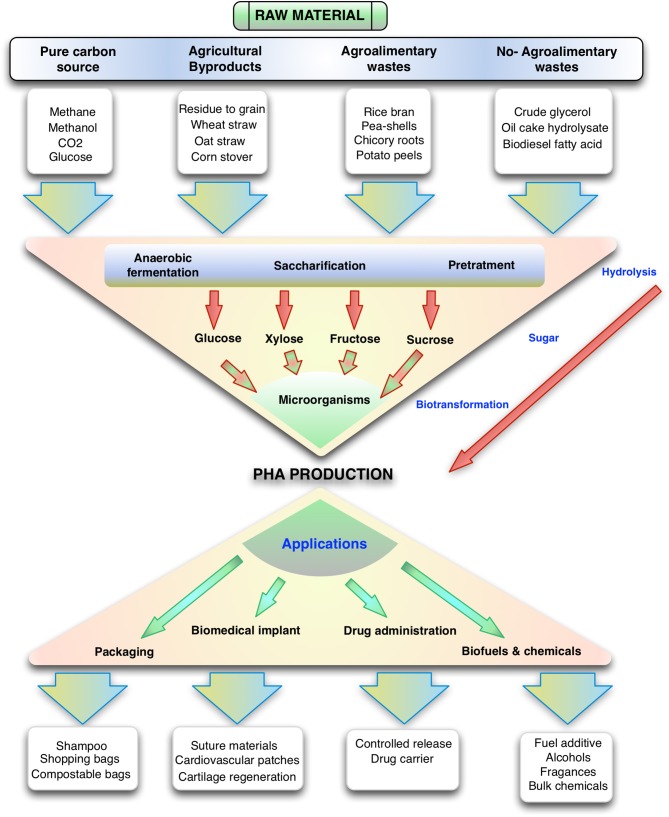
Overview of the production of polyhydroxyalkanoates from different substrates and their uses. Note that many gaseous carbon sources, sugars and other organic molecules can be funneled to this family of polymers.

The most well-known (*R*)-3-hydroxyalkanoic acid, (*R*)-3-hydroxybutyric acid (R3HBA) can be used as a building block in the synthesis of fine chemicals and pharmaceuticals such as antibiotics (Ren et al., 2005), bulk chemicals for the polymer industry (such as hydrogenation to 1,3 butanediol), fragrances and insecticides (Matsuyama et al., 2001) (see Figure 1). 1,3 butanediol can be dehydrated to yield unsaturated alcohols which can be further dehydrated to form 1,3 butadiene (Nozawa et al., 2009), a building block for the production of styrene-butadiene rubber. The world consumption of butadiene reached 10 million metric tons in 2012 (Biddy et al., 2016). R3HBA can be esterified with butanol or ethanol or converted to ethers by reaction with alcohols using the catalytic Williamson ether synthesis (Fuhrmann and Talbiersky, 2005) or dehydrated to crotonic acid, which upon hydrogenation yields butyric acid and *n*-butanol (Schweitzer et al., 2015).

Similarly, 3-hydroxypropionic acid (3HPA), a non-chiral compound, can yield upon chemical modification access to acrylic acid, acrylamide and acrylonitrile, all with market sizes larger than 1 billion dollars, and other niche market compounds such as 1,3 propanodiol, methyl acrylate, and malonic acid (Jers et al., 2019).

More than three decades ago Seebach et al. (1986), reported the synthesis of the macrolide antibiotics pyrenophorin, colletodiol, garamycin A1, and elaiophylidene starting from (*R*)-3-hydroxybutyrate and malate. R3HBA and its derivatives have also been used as potential drugs. Cao et al. (2014) showed that R3HBA and its derivative 3-hydroxybutyrate methyl ester inhibit the development of osteoporosis in mice kept under simulated microgravity. Using *in-vivo* studies with mice suffering from Alzheimer's disease, Zhang et al. (2013) showed that intragastric administration of 3-hydroxybutyrate methyl ester reduced amyloid-β deposition in mouse brains and improved the performance of the treatment group in the Morris water maze (a standardized test in the study of spatial learning and memory) compared to the control group. In a related study in mice, Tieu et al. (2003) showed that the infusion of R3HBA led to improved mitochondrial respiration and ATP production in mice treated with the neurotoxin 1-methyl-4-phenyl-1,2,3,6-tetrahydropyridine causing a mitochondrial deficit reminiscent of Parkinson disease. Finally, Yamanashi et al. (2017) showed that R3HBA could act as a therapeutic candidate for the treatment of stress-related mood disorders (depression). The mechanism involves an anti-inflammatory effect mediated by a reduction in the levels of the inflammatory cytokines inter-leukine 1β and tumor necrosis factor α in the hippocampus of mice.

Other potential uses of R3HBA include its use as a chemical chaperone for heat-mediated denaturation and oxidative damage by Cu^2+^ and H_2_O_2_ produced on industrially relevant enzymes such as lipases and lysozymes (Obruca et al., 2016).

Finally, (*R*)-3-hydroxyalkanoic acids can be used as building blocks that can be biologically produced, excreted from cells and then polymerized or co-polymerized to yield PHAs with the desirable mechanical and thermal properties (Wang et al., 2018). Advantages of the *ex-situ* polymerization starting from biomonomers over the conventional approach of biopolymer accumulation inside cells include (Adkins et al., 2012): (i) simplified downstream product recovery of the extracellular biomonomers compared to the intracellular polymers, (ii) polymerization in controlled chemocatalytic environments allows for polymers with finely tuned properties and high purities, and (iii) higher diversity of plastics thanks to the ability to co-polymerize different monomers. Examples of *in-vitro* synthesis include the production of PHB from R3HBA by a three-enzyme system that only consumes ATP and the monomer (Jossek and Steinbüchel, 1998). The yield of this system was recently improved by using a thermostable acetyl-CoA synthetase, CoA transferase, and PHA synthase (Tajima et al., 2016). A polymer incorporating lactate and R3HBA residues was produced by Tajima et al. (2012) by using an engineered PHA synthase capable of lactate polymerization. An excellent revision of the chemosynthesis of copolymers containing different 3HAs monomers and lactate was recently published (Hiroe et al., 2019). However, a general drawback of these systems so far is the consumption of ATP, specifically to drive the activation of acetate to acetyl-CoA, a molecule that needs to be present on the reaction mixture to act as the CoA donor to yield the hydroxyacyl-CoAs required for the polymerization reaction.

## Substrates for the Production of Polyhydroxyalkanoates and its Monomers

This section summarizes the carbon and energy substrates currently used for the production of PHA and the potential of moving to lignocellulosic substrates, agro-food wastes, and abundant gaseous substrates such as syngas, knallgas, or methane. For a comprehensive review regarding the many substrates for PHA production, the reader is referred to Jiang et al. (2016). The production of 3-hydroxyalkanoic acids from these unconventional sources is presented in section Alternative Substrates and Microorganisms for 3HA Production.

Substrates commonly used for industrial production of PHAs are represented by corn starch, sucrose obtained from sugar cane and vegetable oils, all edible feedstock requiring arable lands and agricultural practices that affect both its economy and sustainability (Levett et al., 2016). Commercial PHAs are produced using sucrose in *Azohydromonas lata* (Biomer Germany, with trade name Biomer^TM^) and *Bacillus* sp. (PHB Industrial S.A. Brazil, Biocycle^TM^); glucose from corn in *C. necator* (Metabolix, Mirela^TM^ and Tianan Biologic Material, Enmat^TM^); or fatty acids using *Pseudomonas putida* (ETH, PHA^TM^) or *C. necator* by Kaneka Corporation and marketed as Kaneka PHBH^TM^ (Chen, 2009; Bugnicourt et al., 2014; Mozejko-Ciesielska and Kiewisz, 2016).

Due to the high costs and sustainability concerns associated with raw materials traditionally used in the production of PHA, the use of agro-food waste, food industry waste, and other non-food industry residues, have been increasingly studied (Braunegg et al., 2004). For example, several solid residues have been examined such as rice bran (Oh et al., 2015), pea-shells (Kumar et al., 2016), chicory roots (Haas, 2015), potato peels, apple pomace, onion peels (Kumar et al., 2016), grape pomace (Follonier, 2015), animal farm waste, poultry litter (Bhati and Mallick, 2016), and palm oil (Loo et al., 2005). In the case of food wastes for PHAs production, literature report on the use of spent coffee grounds (Cruz et al., 2014), food waste composite (Amulya et al., 2015), and used cooking oil (Gómez Cardozo et al., 2016; Borrero-de Acuña et al., 2019).

Finally, non agro-alimentary residues are generated by biodiesel manufacturing: crude glycerol (de Paula et al., 2017), oil cake hydrolysate (Bera et al., 2015), and biodiesel fatty acid by-product from glycerol purification (Cruz et al., 2016). Glycerol can be used for the accumulation of mcl-PHAs and scl-PHAs in *Pseudomonas putida* strains (Poblete-Castro et al., 2014), *C. necator* (Mothes et al., 2007), and *Bacillus megaterium* (Naranjo et al., 2013), among others.

The availability and sustainability issues of conventional substrates have motivated the exploration of alternative feedstock as sources of carbon and energy for microbial production of chemicals, such as lignocellulose which can be obtained from the “residues” left after the harvest of agricultural products or from dedicated high yield cultivars (poplar, eucalyptus, miscanthus) (Loow et al., 2016), thus reducing the environmental burden associated with its production. Reducing even further the water and land usage, compounds containing one carbon atom, such as methanol and formate can be obtained from the reduction of CO_2_ using electrons harnessed from solar energy (Agarwal et al., 2011; Pérez-Fortes et al., 2016; Yishai et al., 2016).

Regarding the use of monomeric sugars obtained from lignocellulose, these are always accompanied by non-sugar compounds like acetic acid, furfural, and other organic acids and aromatic compounds that are produced during pretreatment (Hodge et al., 2008). Biomass is pretreated to improve the action of carbohydrate hydrolases that break down cellulose and hemicellulose during saccharification. In dilute sulfuric acid hydrolysis of corn stover, a leading pretreatment technology, lignocellulosic biomass is deconstructed into pretreated corn stover: a solid fraction (or pulp), enriched in cellulose and lignin, and a liquid fraction (pretreatment liquor), containing oligomers and monomers of hemicellulose and acetic acid (Chen et al., 2012). To further break down cellulose and hemicellulose remaining in the pulp into fermentable sugars, an enzymatic hydrolysis step is necessary.

Regarding PHB production from sugars and acids present in lignocellulosic hydrolysates, albeit *A. lata* or *C. necator* are unable to use xylose, they are tolerant to acetic acid, furfural, and hydroxy-methyl-furfural (among other compounds found in lignocellulosic hydrolysates) (Dietrich et al., 2013). On the other hand, *Burkholderia sacchari* (Brämer et al., 2001) and *Burkholderia cepacia* can use xylose for growth and PHB production. In a fed-batch culture experiment using glucose and xylose to promote PHB accumulation in *B. cepacia*, 60 g L^−1^ of biomass were attained in 70 h with 58% PHB content. The yield of PHB was 0.46 g PHB per gram of substrate (Silva et al., 2004). *B*. *cepacia* was able to incorporate vanillin to PHB (Pan et al., 2012; Dietrich et al., 2013), while levulinic acid was incorporated as hydroxyvalerate (Keenan et al., 2006). Both vanillin and levulinic acid are compounds present in lignocellulosic hydrolyzates. Moreover, the minimum inhibitory concentrations of acetic acid, hydroxy-methyl-furfural and furfural reported for *B. cepacia* growth are 4.00, 6.00, and 6.00 g L^−1^ respectively (Dietrich et al., 2013). Raposo et al. (2017) recently showed that in fed-batch cultures of *B. sacchari* DSM 17165, the catabolite repression of glucose on xylose consumption can be avoided by maintaining glucose concentration below 10 g L^−1^. Notably, they showed that when xylose concentration increases in the fermenter, xylitol appears as a second product (xylose concentration above 30–40 g L^−1^).

Among inexpensive substrates that are readily available for reducing the total cost of PHA production, certain C1 carbon sources, e.g., methane, methanol, and CO_2_ have received a great deal of attention due to their contribution to global warming (Khosravi-Darani et al., 2013). Production of PHB from waste methane may help reduce the impact on the greenhouse effect of this gas. Accumulation of PHB, but not other PHAs, has been studied in several *Methylocystis* species using methane and *Methylobacterium* from methanol [*cf*. Strong et al. (2016)]. In this regard, Listewnik et al. (2007) estimated a price of 6.35 UK pound/kg for produced PHB from natural gas in a two-stage plant producing 500 t PHB per year. Similarly, Levett et al. (2016) estimated a production cost range of 4.1 to 6.8 USD per kg of PHB, with a reduction in the share of the carbon source in the total product cost from 30% when sugar feedstocks are used to 22% for methane. However, these production costs are high when compared to the estimations made by Posada et al. (2011), who performed a techno-economic evaluation of PHB production from glycerol as energy and carbon source. They reported production costs between 1.9 and 2.5 USD per kg and a substrate share between 5 and 8%. The higher production cost when methane is used as substrate is greatly influenced by the slow mass transfer of methane into the media. This leads to large reactors and, thereby high investment costs.

Knallgas bacteria, such as *C. necator* and *A. lata* can use mixtures of hydrogen, carbon dioxide and oxygen for the accumulation of PHB (Reinecke and Steinbüchel, 2008), biofuels (Brigham, 2019), or acetoin (Windhorst and Gescher, 2019). Albeit the production of PHB has been studied thoroughly, with cultures of *C. necator* or *Ideonella* sp. O-1 accumulating over 60 g L^−1^ of PHB (Tanaka et al., 1995, 2011), no reports of PHAs accumulation aside PHB are available (for a review on PHB production from C1 sources see Khosravi-Darani et al., 2013).

## Accumulation and Mobilization of PHAs

The model organism for PHB production is *Cupriavidus necator* (formerly *Ralstonia eutropha* and *Alcaligenes eutrophus*), a gram-negative, obligate aerobe, capable of autotrophic growth in the presence of hydrogen, CO_2_ and oxygen, and heterotrophic growth and PHB production from a wide variety of carbon sources including sugars (chiefly fructose in the wild type organism *C. necator* H16 ATCC 17699) and organic acids (Lu et al., 2016). For example, using glucose in a fed-batch culture of *C. necator* NCIMB 11599, a mutant of *C. necator* H16 capable of using glucose, a concentration of biomass of 164 g L^−1^ was obtained, with a PHB content of 74% (Kim et al., 1994).

In *C. necator*, synthesis of PHB occurs when excess carbon in the form of acetyl-CoA is condensed via a β-ketothiolase (EC 2.3.1.16) to generate acetoacetyl-CoA, which is then reduced to (R)-3-hydroxybutyryl-Coenzyme A by a NADPH-dependent acetoacetyl-CoA reductase (EC 1.1.1.36). Finally, the enzyme PHB synthase (EC 3.1.1.75) catalyzes the polymerization of (R)-3-hydroxybutyrl-CoA monomers. This pathway is the most widespread route in bacteria for providing 3-hydroxybutyryl-CoA monomers (Steinbüchel and Hein, 2001). Production of mcl-PHAs, such as the polymers accumulated in *Pseudomonas* spp., requires precursors derived from the dissociated fatty acid biosynthesis pathway unless these precursors are supplied through related carbon sources (such as octanoic acid for the synthesis of poly(3-hydroxyoctanoate). For details regarding this pathway, we point to the excellent reviews by Lu et al. (2009) and Suriyamongkol et al. (2007).

The regulation of PHB biosynthesis is tightly connected to the cellular levels of reduced nicotinamide nucleotides. Lee (1995) found that when *C. necator* was cultivated in nitrogen-limited media, the NADPH/NADP and NADH/NAD ratios and the intracellular concentrations of NADH and NADP were higher than those found under nitrogen sufficient conditions. Moreover, the rate of PHB accumulation was found to increase with both NADH/NAD and NADPH/NADP ratios. This effect was explained through the analysis of citrate synthase activity. Citrate synthase was inhibited by NADPH and NADH, thus funneling the carbon to PHB instead of being directed to the tricarboxylic acid cycle. Similar conclusions were reported by Henderson and Jones (1997).

Although PHB accumulation occurs under unfavorable conditions for growth induced by nutrient limitation of oxygen, nitrogen, or phosphorous, with excess carbon (Steinbüchel and Hein, 2001), these conditions impact the productivity of the PHB accumulation phase. Grousseau et al. (2013) showed that sustaining a controlled residual growth rate, by feeding a controlled amount of phosphate along with the carbon source (butyric acid), allows for an improved PHB specific productivity and high yield. Interestingly, using Metabolic Flux Balances they showed that the maximal specific PHB production rate is defined by the maximum specific rate of NADPH produced. When a low growth rate is allowed in the fed-batch fermentation (for example, feeding a nitrogen source), the NADPH is produced in the Entner-Doudoroff pathway; whereas without biomass production regeneration of NADPH is only possible via isocitrate dehydrogenase.

Another possibility to increase PHB volumetric productivities is to rely on microorganisms where growth and PHB production occur simultaneously. In this regard, *Azohydromonas lata* consumes glucose, sucrose and acetic acid (Chen et al., 1991), but it does not consume xylose, accumulating PHB during its growth (Yamane et al., 1996). In a fed-batch culture using sucrose as the carbon source and a continuous feeding of ammonia, controlled by the decrease in pH as the culture consumes the source of nitrogen (pH-stat), 143 g L^−1^ of cells with 50% PHB were attained with a PHB productivity of 3.97 g L^−1^ h^−1^, one of the highest ever reported (Yamane et al., 1996). One year later, Wang and Lee (1997) reported even higher productivity (4.94 g L^−1^ h^−1^) and PHB intracellular content in *A. lata* (88%) applying nitrogen limitation in a two-stage fed-batch culture (nitrogen sufficient followed by nitrogen-limited culture).

Assuming that PHAs act as a reserve compound of carbon and energy, without a source of carbon and energy, but in the presence of other growth factors (such as nitrogen or oxygen), PHAs should be depolymerized to its monomers and incorporated into the metabolism, using the degradation products for growth and survival, a process termed PHA mobilization. This behavior has been shown at least in *C. necator* H16 (Uchino et al., 2007; Juengert et al., 2017), *A. lata* (Lee et al., 1999), *Legionella pneumophila* (James et al., 1999)*, Hydrogenophaga pseudoflava* (Choi et al., 1999), and *Halomonas* sp. KM-1 (Kawata et al., 2015).

Compared to PHB accumulation, depolymerization of PHB to R3HBA, and its transformation to acetyl-CoA, has been less studied as confirmed by fewer reports in the literature. However, evidence exists pointing that the granules of PHB and other PHAs are supramolecular complexes (called carbonosomes), constituted by a polymer core and a surface layer of at least a dozen proteins (Sznajder et al., 2015), but without a phospholipids membrane (Bresan et al., 2016). The proteins in the carbonosomes include the PHA synthase (PhaC) and PHB depolimerazes (PhaZs), which are thought to be constitutively expressed (Lawrence et al., 2005; Brigham et al., 2012).

It has been reported that PHB synthesis and its degradation can happen simultaneously in the model PHB accumulating organism *C. necator* (Doi, 1990; Taidi et al., 1995). Doi concluded this after cultivating *C. necator* in butyrate as carbon source under nitrogen-free conditions, thus inducing the accumulation of PHB, and changing the carbon source to pentanoic acid. After the shift in the substrate, the accumulated PHB was gradually replaced by poly(3-hydroxyvalerate-co-3-hydroxybutirate) without a net increase of total polymer content in the cells. This indicates that PHB was degraded and replaced by PHV even in the absence of a nitrogen source. Similarly, Taidi et al. (1995) showed a turnover of PHB after the accumulation of the polymer ceased in a nitrogen-limited batch culture with glucose as the sole carbon and energy source. The turnover was evidenced by the incorporation of radioactivity into the accumulated polymer after feeding labeled glucose (D-[U- ^14^C] glucose). Interestingly, the high-molecular-weight polymer accumulated during the unlabeled glucose phase was replaced by a low-molecular-weight polymer during the labeled glucose experiment. These observations are in agreement with the evidence showing the constitutive expression of PHB synthase and PHB depolymerase in *C. necator* (Lawrence et al., 2005; Sznajder et al., 2015). Corroboration of constitutive expression of PHB synthase and depolymerase in other organisms is scarce, with the exception of the study of Kim et al. (1996) who found simultaneous activities of both enzymes during the batch culture of *Azohydromonas lata* under nitrogen-limited conditions.

Thereby, this leads to the intriguing question of how the synthesis and mobilization of PHAs are regulated, and particularly, how a cycle of simultaneous synthesis and degradation is avoided. Considering that the simultaneous polymerization and depolymerization of PHB and its conversion to acetyl-CoA trough 3-hydroxybutyrate, acetoacetate, and acetoacetyl-CoA requires one NADPH molecule and one ATP molecule and produces only one NADH molecule, the cycle would consume energy for the formation of thioester bonds, thus creating a futile cycle.

To tackle this unsolved issue, Uchino et al. (2007) isolated native PHB granules produced in *C. necator* by glycerol gradient centrifugation to preserve the proteins bounded to the granule, and discovered that in the presence of CoA, these granules produced 3HB-CoA and small amounts of acetyl-CoA. If NAD^+^, but not NADH, is added to the initial reaction mixture, 3HB-CoA remains undetectable, but the concentration of acetyl-CoA increases 5-fold. The authors assumed that in the presence of NAD^+^, the intermediately formed 3HB-CoA is rapidly transformed to acetyl-CoA in NAD^+^ dependent reactions. The authors also found, as expected, that the native granules release R3HBA in pH-stat experiments using methods previously described (Gebauer and Jendrossek, 2006). Therefore, it remains unclear which depolymerization mechanism, hydrolysis or thiolysis, is used *in-vivo* for the mobilization of PHB.

In order to identify the enzymes responsible for the thiolytic cleavage, Uchino et al. (2007) incorporated the PHB synthesis genes *phaCAB* in *E. coli* S17-1 along with the phasin gene *phaP1*, the depolymerase gene *phaZa1* or *phaP1* + *phaZa1*. Interestingly, the recombinant strain only produced 3HB-CoA in the presence of CoA when both *phaP1* and *phaZa1* were present in a *phaCAB* background. Moreover, no significant amounts of acetyl-CoA were detected in this experiment, an indication that downstream enzymes for the use of 3HB-CoA were absent in *E. coli*.

**Figure 2:**
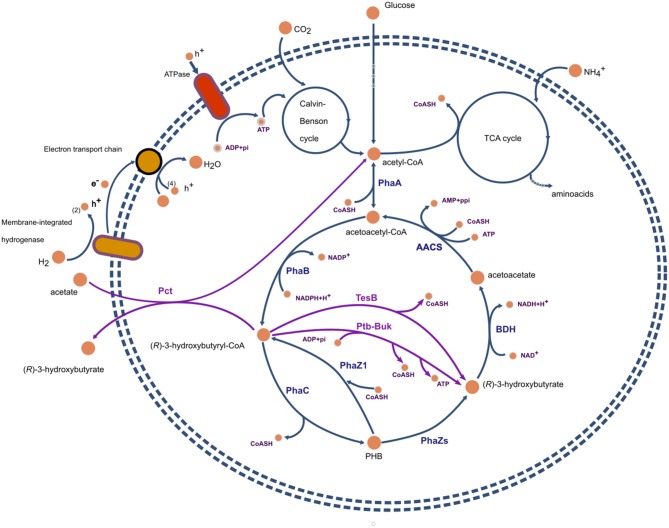
Representation of metabolic pathways involved in the synthesis of polyhydroxybutyrate and (*R*)-3-hydroxybutyrate in *Cupriavidus necator*. Reactions and proteins present in the wild type are presented in blue. In magenta, recombinant proteins and reactions commonly expresed in host organisms such as *E. coli*. PhaA, β-ketoacyl-CoA thiolase; PhaB, acetoacetyl-CoA reductase; PhaZs, PHA depolymerases; BDH, (*R*)-3-hydroxybutyrate dehydrogenase; PhaC, PHA polymerase; TesB, acyl-CoA thioesterase II; Ptb-Buk, phosphor-transbutyrylase and butyrate kinase; AACS, acetoacetyl-CoA synthase.

The study of Uchino et al. (2007), suggests that *in-vivo* intracellular depolymerization of PHB does not represent a futile cycle and an energy waste in the form of thioester bonds. If the main product of PHB polymerization, at least in *C. necator*, is 3HB-CoA instead of 3HB, then there is no loss of energy for the formation of acetoacetyl-CoA from acetoacetate (see Figure 2).

Regarding the regulation of PHB synthesis and degradation, Juengert et al. (2017), found that the degradation of the accumulated PHB in *Ralstonia eutropha* was fast and efficient in the absence of the alarmone (p)ppGpp, and that when present, (p)ppGpp directly or indirectly inhibits PHB mobilization. (p)ppGpp is a key signaling molecule, which, when present at high concentrations induces the stringent response in *E. coli* and other bacterial species. This alarmone accumulates in amino acids starved cells, and inhibit the synthesis of ribosomal and transfer RNAs (Srivatsan and Wang, 2008). The results suggest that PHB accumulation under nitrogen-limited conditions is favored by the inhibition of PhaZ1 by (p)ppGpp as shown by the observed PHB accumulation in a triple knockout mutant (Δ*spoT*1+Δ*spoT*2+Δ*phaZa*1) that was unable to produce (p)ppGpp. In contrast, the double knockout mutant Δ*spoT*1+Δ*spoT*2 accumulated negligible amounts of PHB. In a subsequent work, Juengert et al. (2018) identified that PhaC1 was phosphorylated in multiple phosphosites during the stationary growth phase in nutrient broth medium with gluconate as carbon source, but it was not modified during the exponential and PHB accumulation phases or when grown in a fructose-mineral medium. On the other hand, PhaZa1 was phosphorylated in Ser35 both during the exponential and stationary growth phases. Mutagenesis of the identified residues showed that PHB accumulation was unaffected for most mutants of PhaC1, except for a mutant with changes to four aminoacid residues. On the other hand, exchanging the phosphorylated residues in PhaZa1 to aspartate (a phosphomimetic residue) produced mutants with a strongly reduced ability to mobilize the accumulated PHB.

Little experimental evidence exists concerning the role of the stringent response in the accumulation of PHA in other organisms. In this regard, Mozejko-Ciesielska et al. (2017) obtained a *relA*/*spot* mutant of *P. putida* KT2440. This mutant, unable to induce the stringent response, was used to assess the accumulation of PHA under nitrogen deprived and optimal nitrogen conditions. Results show that this mutant is able to accumulate mcl-PHAs under both conditions.

Taken together, the works of Mozejko-Ciesielska et al. (2017) and Juengert et al. (2017; 2018) provide insights into the molecular basis of the regulation of PHB accumulation and mobilization. As it will be covered in section *in-vivo* Strategies these regulatory mechanisms play a key role in the production of R3HBA *in-vivo* from cells that accumulated PHB.

## Production of R-3- Hydroxyalkanoic Acids From PHAs

Several strategies based on operational changes or genetic modifications have been devised for the production of chiral hydroxyalkanoic acids. This section organizes such strategies in three categories, *ex-vivo* strategies, *in-vivo* strategies using non-genetically modified organisms and, finally, the construction of recombinant organisms. This material updates and complements the excellent review of Ren et al. (2010), that extensively covers the potential applications of hydroxyalkanoic acids and also focused on the chemical synthesis of these compounds and the review by Gao et al. (2011), dealing with a broader view, the synthesis of PHAs and its monomers.

### *In-vitro* Strategies

Under this approach, the accumulated PHAs are extracted from cells and then subjected to a chemical or enzymatic depolymerization process. The PHA recovery process starts when cells are separated by centrifugation to increase product concentration and to remove the components of the culture media. The concentration step is followed by drying or lyophilization of the concentrated biomass. The extraction of PHAs from the dried biomass can be performed by solvent extraction, by dissolving the biomass using oxidizing agents or by disrupting cells to liberate the PHAs granules (Kosseva and Rusbandi, 2018). Mechanical cell disruption techniques, such as bead milling and high-pressure homogenization have been widely used to release intracellular protein, and have been adapted for PHAs recovery (Melih Tamer et al., 1998; Kunasundari and Sudesh, 2011). Melih Tamer et al. (1998) compared bead milling and high-pressure homogenization for the recovery of PHB accumulated in *Azohydromonas lata*. They recommended bead milling over high-pressure homogenization as the preferred method for recovering PHB from heat-shocked cells of *A. lata*. Interestingly, since PHB is recovered without solubilizing it, the native amorphous morphology of the polymer was conserved.

The common method for PHAs extraction is the use of solvents for its speed and simplicity. Solvents alter the cell membrane and then dissolve the polymers. PHAs are recovered by solvent evaporation or precipitation with an anti-solvent (Liddell, 1999; Hänggi, 2012). In the solvents category, the chlorinated hydrocarbons chloroform, 1,2-dichloroethane, and methylene chloride are used (Ramsay et al., 1994). Non-chlorinated solvents have been proposed such as ethylene carbonate and 1, 2-propylene (Lafferty and Heinzle, 1977) or solvent mixtures of acetone/ethanol/propylene carbonate (Fei et al., 2016). On the other hand, the precipitation of PHAs is generally induced by non-solvent agents such as water, ethanol, and methanol (Ramsay et al., 1994; Mikkili et al., 2014). In summary, solvent extraction is characterized by numerous advantages such as the elimination of endotoxins and low polymer degradation (Liddell, 1999). However, there are hurdles in the use of solvents, including their high cost (Poirier et al., 1995), high energy consumption for the separation of miscible solvents and anti-solvents, and risks both for the operator and for the environment that need to be considered during the process design stage (Gorenflo et al., 2001).

On the other hand, digestion with sodium hypochlorite decomposes the cells allowing high levels of PHA purity to be reached (Hahn et al., 1994; Kim et al., 2003), however, sodium hypochlorite degrades PHB, resulting in a polymer with low molecular weight (Ramsay et al., 1990; Mikkili et al., 2014).

#### Chemical and Enzymatic Hydrolysis of Recovered PHAs

After recovery, and possible purification, the extracted PHAs can be chemically or enzymatically converted into high-purity 3-hydroxyalkanoic acids. Chemical methods for modifying PHB have been reported for the production of (*R*)-methyl 3-hydroxybutanoate and (*R*)-3-hydroxybutanoic acid, these methods involve the use of methanol and sulfuric acid and methanol and *p*-toluenesulfonic acid monohydrate respectively (Seebach et al., 2003). Another approach, reported by Lee et al. (2000), considers the acidic alcoholysis of PHB, yielding methyl, ethyl, and *n*-propyl esters of (*R*)-3- hydroxybutyrate, using 1,2 dichloroethane as solvent with either sulfuric acid or hydrochloric acid as catalyst. de Roo et al. (2002) extended this method for the production of mcl-3HAs from the mcl-PHAs accumulated in *P. putida*. After a step of acid methanolysis, the obtained (*R*)-3HA methyl esters were distilled into fractions and saponified to yield the corresponding (*R*)-3-hydroxycarboxylic acids.

The multistep chemical processes outlined in the previous paragraph can be efficiently catalyzed by PHA depolymerases in a single step. This subject has been recently reviewed (Roohi and Kuddus, 2018). Thus, only some selected works will be covered. Several extracellular depolymerases have been identified and characterized (an excellent tool for its classification was presented by Knoll et al., 2009). Extracellular PHA depolymerases degrade denaturated PHA granules whose structure has been altered during the extraction process and are not covered by the native layer of proteins surrounding the granule. One exception is the PHB depolymerase from *Pseudomonas lemoignei* which is active against native PHB granules (Handrick et al., 2001).

PHB degradation studies by extracellular depolymerases are typically performed using PHB films as substrate, producing R3HBA as the hydrolysis product (Polyák et al., 2018), thus limiting the access of enzymes to its substrate. In order to achieve high R3HBA titers, a high initial concentration of PHB should be used. However, due to its water-insoluble nature, only low PHB concentrations can be suspended in water. For example, 24 g L^−1^ of 3RHBA were produced from 25 g L^−1^ of suspended PHB powder using an extracellular PHB depolymerase from *Pseudomonas* sp. DS1001a (Li et al., 2016). As with chemical hydrolysis methods, enzymatic hydrolysis requires the separation and partial purification of the PHA accumulated in cells and this contributes to the overall costs of 3HAs. These disadvantages could be eliminated in processes where 3HAs are produced directly from the PHAs accumulated by the cells using their own intracellular enzymes for the depolymerization process.

### *In-vivo* Strategies

#### Hydroxy-Acids Production by *in-vivo* Depolymerization of PHAs

This section reviews the production of 3HAs obtained through the depolymerization of PHAs accumulated using sugars and fatty acids as carbon and energy sources. The analysis of 3HAs obtained from other carbon sources is deferred to section Alternative Substrates and Microorganisms for 3HA Production.

*In-vivo* depolymerization of accumulated PHAs to R3HAs has been reported to occur with high yields in *Azohydromonas lata* (Lee et al., 1999) and *Pseudomonas putida* GPo1 (Ren et al., 2005). The depolymerization process has been shown to be highly dependent on pH. Lee et al. (1999), in a pioneering work, achieved the depolymerization of PHB to R3HBA in *A. lata* cells grown in mineral medium with sucrose as the carbon and energy source. The depolymerization process was carried out at different initial pHs in water, after washing the cells, at 37°C and without shaking to minimize oxygen transfer. Exceedingly high R3HBA yields and productivities, as high as 96% in 30 min were found at pH 4.0, but not at higher pH values. This result was explained in terms of the effects of pH on PHB depolymerase and 3-hydroxybutyrate dehydrogenase activities. The highest activity of PHB depolymerase was achieved at pHs 3 and 4, at which the monomer production rate was also the highest. Interestingly, in *A. lata* no activity of (*R*)-3-hydroxybutyric acid dehydrogenase was detected at pH 4, therefore it was argued that no 3HBA was degraded to acetoacetate (see Figure 2 for the degradation pathway).

At pH 5, the attained depolymerization yield was 31% and decreased toward neutral pHs. From the work of Lee et al. (1999), it is not possible to ascertain whether these lower yields are due to the consumption of the released R3HBA or a decrease in the amount of PHB depolymerized. Presumably the latter is true since the depolymerization assays were performed without shaking, thus restricting the ability of cells to regenerate NADH into NAD^+^, a cofactor of 3-hydroxybutyrate dehydrogenase.

Following the high depolymerization yields obtained at low pHs, the authors assayed the *in-vivo* depolymerization in *Cupriavidus necator* NCIMB 11599, *Pseudomonas oleovorans* ATCC 29347, and *Pseudomonas aeruginosa* PAO1 (DSM 1707) at pH values below 7.0. The depolymerization yields and productivities were close to 20% for *C. necator* and below 10% for *Pseudomonas* species (see Table 1).

**Table 1:** Summary of studies reporting the production of 3HA in non-genetically modified organisms using several operational strategies.

**Microorganism**	**Strategy**	**Hydroxy acid^**d**^**	**Yield ^**a**^(3HA Titer, gL^**−1**^)**	**HA Volumetric productivity (gL**^****−1****^ **h**^****−1****^**)**		**References**
				**Depolymerization^**b**^**	**Fermentation and depolymerization^**c**^**	
*Azohydromonas lata* DSM 1123	Water, initial pH 4.0, 37°C	R3HBA	84% (117.8)	117.8	4.91	Lee et al., 1999
			96% (0.99)	1.98	0.0825	
*Cupriavidus necator* NCIMB 11599	Water, initial pH 7.0, 30°C	R3HBA	19% (5.8)	0.17	NA	Lee et al., 1999
		R3HVA	23% (0.6)	0.017	NA	
*Pseudomonas oleovorans* ATCC 29347	Water, initial pH 7.0, 30°C	R3HHx	9.2% (0.13)	0.0014	NA	Lee et al., 1999
		R3HO	9.7% (1.42)	0.015	NA	
*Pseudomonas aeruginosa* PAO1 (DSM 1707)	Water, initial pH 7.0, 30°C	R3HO	9.6% (0.34)	0.0035	NA	Lee et al., 1999
		R3HD	8.8% (1.02)	0.0106	NA	
		R3HDD	6.7% (0.08)	0.0008	NA	
*P. putida* GPo1	50 mM potassium phosphate buffer, pH 11, 30°C	R3HO	76%(0.356)	0.059	0.022	Ren et al., 2005
		R3HHx	21%(0.015)	0.003	0.001	
*P. putida* GPo1	50 mM potassium phosphate buffer, pH 10, 30°C	mcl-HAs	Average 70% (≈ 1.1)	0.14	0.058	Ruth et al., 2007
*P. putida* GPo1	Culture broth, pH-stat at pH 10, 30°C.	R3HOR3HHx	90% (0.63)	NA	0.042	Ren et al., 2007
*P. putida* Bet001	0.2MTris–HCl buffer, pH 9, I = 0.2M, 30 °C	R3HO	54% (0.06)	0.001	0.001	Anis et al., 2018
		R3HHx	69% (0.64)	0.013	0.007	
		R3HD	98% (0.73)	0.015	0.008	
		R3HDD	47% (0.18)	0.004	0.002	
*Halomonas* sp. KM-1	Shift to microaerobic conditions under nitrogen rich condition	R3HBA	55% (40.3)	1.68	0.48	Kawata et al., 2014
*Halomonas* sp. OITC1261	Aerobic culture, sucrose, sodium nitrate as limiting nutrient	R3HBA	58 g L^−1^ R3HBA +27 g L^−1^ PHB	NA	0.65	Yokaryo et al., 2018

a Yield refers to the mass of 3-hydroxyalkanoic acid obtained over the initial mass of polyhydroxyalkanoates in cells mass. The titer of 3HA is shown in parenthesis.

b Volumetric productivity of the depolymerization process, not accounting for the time required for PHAs production.

c Volumetric productivity of the depolymerization and fermentation process, accounting for the time required for PHAs production.

d *R3HBA, (R)-3-hydroxybutyric acid; R3HVA, (R)-3-hydroxyvaleric acid; R3HHx, (R)-3-hydroxyhexanoic acid; R3HO, (R)-3-hydroxyoctanoic acid; R3HD, (R)-3-hydroxydecanoic acid; R3HDD, (R)-3-hydroxydodecanoic acid*.

Ren et al. (2005) showed that for *Pseudomonas putida* GPo1, the PHB depolymerization rate was higher at pH 11 in citrate buffer, which helps to control the pH drop caused by the accumulation of R3HAs. The released monomers corresponded to (*R*)-3-hydroxyoctanaoic (R3HO) acid and (*R*)-3-hydroxyhexanoic acid (R3HHx), in a proportion closely matching the ratio of monomers in the copolymer accumulated in continuous culture with octanoic acid as carbon and energy source. The depolymerization was performed for 6 h, thus decreasing the volumetric productivity compared to the work of Lee et al. (1999) (see Table 1). A second factor decreasing the volumetric productivity was the low initial PHA concentration used. However, this work showed that R3HAs different than R3HBA could be obtained with high yields by using the correct pH during the depolymerization process. This work was further extended by Ruth et al. (2007) showing that applying the same depolymerization process at pH 10 to PHAs accumulated in *P. putida* GPo1, grown in continuous culture with either octanoic, undecanoic or 10-undecenoic acid, led to the production of (*R*)-3-hydroxyoctanoic acid (R3HO), (*R*)-3-hydroxyhexanoic acid (R3HHx), (*R*)-3-hydroxy-10-undecenoic acid (R3C11-1), (*R*)-3-hydroxy-8-nonenoic acid (R3C9-1), (*R*)-3-hydroxy-6-heptenoic acid (R3-C7-1), (*R*)-3- hydroxyundecanoic acid (R3-C11-0), (*R*)-3-hydroxynonanoic acid (R3-C9-0), and (*R*)-3-hydroxyheptanoic acid (R3-C7-1).

Recently, Anis et al. (2018) studied the *in-vivo* depolymerization of PHAs accumulated in *P. putida* Bet001 after 48 h of batch culture with lauric acid as the carbon source and under nitrogen-limited conditions. The depolymerization was performed for 48 h in 0.2M Tris–HCl buffer, pH 9, and 30 °C. Unlike the report of Lee et al. (1999) using *Pseudomonas aeruginosa* PAO1 (DSM 1707), *P. putida* Bet001 produced R3HO, R3HHx, (*R*)-3-hydroxydecanoic acid (R3HD), and (*R*)-3-hydroxydodecanoic acid (R3HDD) with very different yields (see Table 1), being the highest depolymerization yield achieved for R3HD. It is not clear if this difference in yields is due to a channeling of R3HO, R3HHx, and R3HDD toward cell metabolism or if they have yet to be hydrolyzed from the granules and thus, reflects an affinity of the PHA depolymerases.

The experiments performed by Lee et al. (1999), Ren et al. (2005), and Ruth et al. (2007) used either water or phosphate buffer as depolymerization media at a pH initially set at a given value. However, the release of 3HAs leads to a decrease in the initial pH, potentially affecting process efficiency. This factor was recognized by Wang et al. (2007), leading to the design and application of a pH-stat process. In this system, the pH is controlled at a setpoint by the automatic addition of an alkaline solution (NaOH). Interestingly, the amount of NaOH added in time (the flow of the alkaline solution), if recorded, allows the estimation of the release rate of acids (hydroxy acids and protons). Using a pH-stat apparatus coupled to a dissolved oxygen meter, Wang et al. (2007) investigated the behavior of the wild type *P. putida* GPo1 strain and a PHA depolymerase negative mutant. Results showed that the rate of acid production (not necessarily hydroxy acids) of the mutant strain was only 27% of the rate obtained with the wild type. Analysis of the supernatants revealed that the acids released by the wild type were R3HO and R3HHx. On the other hand, no detectable amounts of these compounds were found in the supernatants of the depolymerase mutant. Moreover, oxygen consumption measurements indicated a low respiratory activity for the wild type and a high respiration rate for the mutant. Finally, they also found that the acid production rate of the mutant, but not of the wild type, could be enhanced by aeration. These results support the hypothesis that the high depolymerase activity allowed the wild type strain to compensate for the high external pH. On the other hand, in the PHA depolymerase deficient mutant this could only be performed by the production of protons in aerobic conditions.

Although the results obtained by Wang et al. (2007) support this hypothesis for the depolymerization of PHAs to 3HAs at high pHs, the compensatory mechanism of pH involved in it does not explain the behavior found for *A. lata* at low pH values. Thus, the only conclusion applicable for both species is that in *A. lata* and *P. putida* GPO1 the depolymerization process is enhanced at a certain pH and simultaneously the consumption of the released monomers is impaired.

Process engineering strategies for 3HAs production are nearly non-existent with the exception of the work of Ren et al. (2007) who coupled a chemostat culture of *P. putida* GPo1 (nitrogen-limited, dilution rate of 0.1 h^−1^, octanoic acid as the sole carbon source) to a second continuous depolymerization stirred tank reactor. The depolymerization tank was operated as a pH-stat at a pH of 10.0 and its discharge was sent to a plug-flow reactor with a residence time of 6 h. This continuous system does not require the separation of cells or the exchange of the culture media to water or buffer. Only a simple pH shift was enough to achieve a depolymerization yield of 90%, however, the volumetric productivity was not different from previously reported works using batch depolymerization (Ren et al., 2005). Clearly, the volumetric productivity could be improved by increasing the concentration of PHA leaving the chemostat.

A different strategy for the *in-vivo* production of R3HBA has been reported using several strains of *Halomonas*. Using *Halomonas* sp. KM-1, 15.2 g L^−1^ of R3HBA could be obtained under microaerobic conditions from 16.4 g L^−1^ of PHB that were accumulated under aerobic conditions using glycerol as the sole carbon and energy source. The initial concentration of the nitrogen source was 12.5 g L^−1^ (sodium nitrate), hence the limiting nutrient was presumably different from nitrogen, although this was not clarified (Kawata et al., 2012). These conditions, when applied to cultures grown in glucose (Kawata et al., 2014) did not result in any 3HBA secretion. The glucose concentration decreased from 20 to 6% during the first 36 h of culture and then remained constant. Thus, in this experiment, a nitrogen shortage was suspected.

When extra nitrate was pulse fed at 24, 36, and 48 h to a culture of *Halomonas* sp. KM-1 with 20% (w/v) glucose, then 40.3 g L^−1^ R3HBA were secreted with a productivity of 0.48 g L^−1^ h^−1^ after a shift from aerobic cultivation for 60 h to microaerobic cultivation for 24 h. No R3HBA was secreted when no extra nitrate was supplemented (Kawata et al., 2014) indicating that a regulatory mechanism was controlling the activity of the PHB depolymerase, presumably related to the stringent response (see section Accumulation and Mobilization of PHAs). Glucose concentration during the depolymerization phase under microaerobic conditions was zero and a decrease in total cell concentration was observed. This behavior is consistent with the depolymerization of the accumulated PHB with only partial use of the released R3HBA for growth (our calculations show that 79% of the depolymerized PHB was recovered as 3HBA, equivalent to 55% of the maximum recovery if all the accumulated PHB is transformed to 3HBA). This can be ascribed to the microaerobic conditions applied that limited the use of acetyl-CoA for growth and the regeneration of NAD^+^ from NADH. Since NAD^+^ is the cofactor used by 3-hydroxybutyrate dehydrogenase, this could explain the high titers of R3HBA. Unfortunately, no nitrate concentrations during the growth or depolymerization phase were reported to ascertain whether it is consumed or not during the microaerobic cultivation. Moreover, PHB was not completely mobilized. Since no information regarding the pH of the culture (or its control) was presented, presumably the lack of complete depolymerization was caused by a decrease in pH.

A new species of *Halomonas, Halomonas* sp. OITC1261 was isolated by Yokaryo et al. (2018). Unlike *Halomonas* sp. KM-1, *Halomonas* sp. OITC1261 produces R3HBA under aerobic conditions and, apparently, without the need to supplement with extra nitrogen source once the carbon source is exhausted to promote PHB depolymerization. In fact, the data presented by Yokaryo et al. (2018) showed that R3HBA is produced concomitantly with PHB after ~10 h of cell growth. Presumably, PHB and R3HBA started to accumulate after the exhaustion of the nitrogen source, which could also explain the increase in the dissolved oxygen concentration. It is not clear whether the production of R3HBA occurs through PHB *in-vivo* depolymerization or proceeds directly from (*R*)-3-hydroxybutyryl-CoA or acetoacetate.

#### Production of Hydroxyalkanoates in Genetically Modified Microorganisms

##### Mutants of native PHA producers

The exploration of alternative pathways for the production of R3HAs emerges from the recognition of two characteristics found in native PHA producers: (i) depolymerization products can be metabolized, for example, R3HBA is converted to acetoacetate by the 3-hydroxybutyrate dehydrogenase (HBD) enzyme (Tokiwa and Ugwu, 2007) and (ii) producing 3HA is a two-stage process where PHA is accumulated and then depolymerized in a subsequent step that often requires a change in media or process conditions. A process for the isolation of 3-hydroxybutyrate dehydrogenase null mutants was described more than 40 years ago (Lafferty, 1977), including UV mutagenesis, followed by the destruction using antibiotics of the bacteria capable of 3HBA assimilation and the selection of 3HBD null mutants by spread plating of the individuals surviving the treatment with bactericidal.

Ugwu et al. (2008), reported the production of R3HBA in *C. necator* through the acetoacetate pathway induced by random mutation using UV radiation. The mutants achieved a titer of 0.150 g L^−1^ of R3HBA in a 5 L fermenter after 48 h of cultivation using either glucose or sucrose as carbon source. The concentration of R3HBA was increased by feeding lithium acetoacetate to resting cells of the mutant strain, reaching 0.84 g L^−1^ of R3HBA. The results were interpreted as indicative of a disruption in the *phbB* gene (coding for acetoacetyl-CoA reductase), making this strain unable of PHB accumulation (see Figure 2). Ugwu et al. (2008) reasoned that the excess of acetoacetyl-CoA produced under conditions allowing for PHB accumulation was channeled toward R3HBA via acetoacetate.

Using UV mutagenesis, Ugwu et al. (2011) obtained an R3HBA-producing mutant of *Azohydromonas lata*. When cells of this mutant were resuspended in phosphate buffer containing glucose (1% v/w), ethylacetoacetate (2% v/v), or (R,S)-1,3-butanediol (3% v/v), the resting cells produced R3HBA at concentrations of 6.5, 7.3, and 8.7 g L^−1^, respectively.

##### Production of 3HAs in recombinant E.coli strains

 </H4>An alternative process for the production of R3HBA is the use of recombinant methods to express PHA related genes in well-characterized non-PHA producing and fast-growing microorganisms such as *Escherichia coli* (Chen et al., 2013). Lee et al. modified *E. coli* strains by inserting two plasmid systems containing the *phaA*_*Re*_, *phbB*_*Re*_, and *phbC*_*Re*_ genes) and the *phaZ1*_*Re*_ depolymerase from *Ralstonia eutropha* (Lee and Lee, 2003). This design achieved an R3HBA concentration of 9.6 g L^−1^ in 51 h of fermentation using glucose as a carbon source.

Shiraki et al. (2006) engineered *E. coli* and *R. eutropha* to express the same enzymes leading to the production of PHB but different depolymerases. The strains compared were an *R. eutropha* null mutant for 3-hydroxybutyrate dehydrogenase and a recombinant *E. coli* strain harboring the synthetic PHB operon of *R. eutropha* and an extracellular depolymerase of *Paucimonas lemoignei*. The production of R3HBA by the 3-hydroxybutyrate dehydrogenase null mutant of *R. eutropha* was found to be dependent on the supply of oxygen, achieving an R3HBA concentration of 3.13 g L^−1^ under anaerobic conditions and concentrations in the 0.41–1.04 g L^−1^ range under aerobic culture conditions. The intracellular PHB content decreased concomitantly with the production of R3HBA, reaching almost zero for the experiment performed under anaerobic conditions after 50 h of depolymerization. Presumably, under aerobic conditions the accumulated PHB was depolymerized to a molecule different than R3HBA, such as 3-hydroxybutyryl-CoA, or the produced R3HBA was metabolized using pathways not using 3-hydroxybutyrate dehydrogenase (BDH). In fact, Shiraki et al. (2006) verified that no BDH was expressed and no BDH was found in the supernatant fraction of cells grown under PHB accumulation conditions, however, it was not verified whether the mutant could grow in R3HBA. On the other hand, the recombinant *E. coli* harboring the PHB operon and the *P. lemoignei* depolymerase reached a concentration of approximately 7.3 g L^−1^ of R3HBA after 100 h. Since *E. coli* is not a native PHB producer, presumably there were no alternative pathways for R3HBA production or consumption different from the inserted genes (Shiraki et al., 2006). Interestingly, the difference between the R3HBA production pathways used by Shiraki et al. (2006) and Lee and Lee (2003) is the PHB depolymerase gene. While Shiraki used the extracellular depolymerase of *Paucimonas lemoignei* capable of hydrolyzing both native PHB granules and denaturated ones (Handrick et al., 2001), Lee and Lee used a gene from *R. eutropha*. In particular, the depolymerase PhaZa1 used by Lee and Lee (2003) was found to secrete rather low concentrations of R3HBA when expressed in the same *E. coli* strain (Uchino et al., 2008).

Generally, the use of the *phaCAB* and *phaZ* genes requires a two-stage fermentation, accumulation of the polymer followed by depolymerization, thereby the required cultivation times are usually long. Hence, the interest in the last decades focused on the direct microbial production of extracellular 3HA monomer without going through these two stages and achieving a straightforward, less energy and time-consuming process.

The first alternative pathway was reported by Gao et al. (2002), by introducing the *phbA, phbB, buk* and *ptb* genes in recombinant *E. coli* DH5K. This pathway starts from glucose to yield acetyl-CoA, which is the key compound in glycolysis, PhbA catalyzes the formation of acetoacetyl-CoA from two acetyl-CoAs, which is then reduced to (*R*)-3-hydroxybutyryl-CoA under the action of PhbB. (*R*)-3-hydroxybutyryl-CoA is then transformed into (*R*)-3-hydroxybutyryl-P by Ptb (see Figure 2). Finally, Buk converts the (*R*)-3 hydroxybutyryl-P into R3HBA, resulting in the production of 12 g L^−1^ in fed-batch cultures (Gao et al., 2002). The direct method presents some advantages: (i) glucose is transformed without accumulation and depolymerization of PHB, (ii) R3HBA production is not associated with cell growth, and (iii) immobilization of the recombinant strain may allow a continuous R3HBA production process to be established.

Similarly to the system presented by Gao et al. (2002), (*R*)-3HB-CoA can be directly hydrolyzed into R3HBA using TesB, a class II thioesterase enzyme that catalyzed the hydrolysis of the CoA moiety from acyl-CoAs (Naggert et al., 1991). For example, when TesB acts on 3 hydroxybutyryl-CoA, free R3HBA monomers are obtained by cleaving coenzyme A.

Liu et al. (2007) used *E. coli* BW25113 as host for the expression of *phbA* and *phbB* genes from *R. eutropha* and *tesB* from *E. coli*. This strain produced 3.98 g L^−1^ R3HBA in shake flask culture using 11.4 g L^−1^ of glucose. Using a fed-batch strategy, 12.2 g L^−1^ R3HBA were accumulated in 24 h. The feeding strategy consisted of pumping a solution containing glucose (20 g L^−1^) and ammonium sulfate (2 g L^−1^) each time glucose concentration in the broth was lower than 10 g L^−1^. The productivity achieved by this system (0.51 g L^−1^ h^−1^) is among the highest reported so far for the direct production of R3HBA (see Table 2). Previous to this work, it was well established that TesB was capable of releasing CoA from acyl CoA of C_6_-C_18_ carbon length, as well as 3-hydroxyacyl-CoA esters, to their corresponding free fatty acids (Naggert et al., 1991), but not from short-chain length hydroxyacyl-CoA. In this regard, Zheng et al. (2004) reported the production of (*R*)-3-hydroxydecanoic acid (R3HD) from fructose by a recombinant *E. coli*. The recombinant strain contains the *phaG* gene from *P. putida* encoding for (*R*)-3-hydroxydecanoyl-acyl carrier protein-coenzyme A transacylase. PhaG links fatty acid *de novo* biosynthesis and PHA production by catalyzing the conversion between (*R*)-3-hydroxydecanoyl-acyl carrier protein and (*R*)-3-hydroxydecanoyl-CoA (Rehm et al., 1998). When the *E. coli* strain containing only PhaG was cultured in shake flasks with 20 g L^−1^ fructose, 0.64 g L^−1^ of R3HD, and 2.11 g L^−1^ of biomass were obtained. The R3HD titer was increased to 1.02 g L^−1^ when a plasmid expressing *tesB* was also inserted into the strain along with *phaG*, suggesting that the activity of TesB in the strain containing only *phaG* was insufficient for efficient hydrolysis of the produced (*R*)-3-hydroxydecanoyl-CoA.

**Table 2:** Summary of engineered strains for the production of 3-hydroxyalkanoic acids.

**Strains**	**Carbon substrate**	**3HA titer (gL^**−1**^)^**a**^**	**3HA Volumetric productivity (gL^**−1**^ h^**−1**^)**	**References**
*E. coli* DH5α(phbA-phbB-buk+ ptb)	Glucose	12.0 (R3HBA)	0.25	Gao et al., 2002
*E. coli* XLI-Blue (phaA-phaB-phaC-phaZi)	Glucose	9.6 (R3HBA)	0.19	Lee and Lee, 2003
*E. coli* DH5 (phbA-phbB-phaG)	Glucose + Acrylic acid	0.7 (R3HBA)	0.01	Zhao et al., 2003
*E. coli* JM 109 (pha CAB-phaZ7)	Glucose	7.3 (R3HBA)	0.073	Shiraki et al., 2006
UV radiation mutant *Cupriavidus necator*	Glucose or sucrose + Lithium acetoacetate	0.84 (R3HBA)	0.026	Ugwu et al., 2008
UV mutant radiation *Azohydromonas lata*	Sucrose +1,3 butanodiol	8.7 (R3HBA)	0.082	Ugwu et al., 2011
*E. coli* BW25113 (phbA- phbB - tesB)	Glucose	12.2 (R3HBA)	0.51	Liu et al., 2007
*Pseudomonas putida* KTOY07 (with pSPH09 plasmid)	Lauric acid	7.27 (96% R3HDD)	0.26	Chung et al., 2009
*E. coli* K-12 MG1655(DE3)	Glucose	2.92 (R3HBA)	0.06	Tseng et al., 2009
		2.08 (S3HBA)	0.04	
*E. coli* with the plasmid sets pET-PB-B, and pCDF-T-H (for (S)-3HV synthesis) or pCDF-T-P (for (R)-3HV synthesis)	Glucose	0.50 (R3HVA)	0.007	Tseng et al., 2010
		0.31 (S3HVA)	0.004	
	Glycerol	0.60 (R3HVA)	0.0084	
		0.19 (S3HVA)	0.003	
*E. coli* (phaAB-pct)	Glucose +acetate	5.2 (R3HBA)	0.22	Matsumoto et al., 2013
Mutant of *M. rhodesianum* (DlipA^−^ Dhbd^−^)	Methanol	2.81(R3HBA)	0.014	Hölscher et al., 2010
*E. coli* strain AF1000 pJBGT3RX	Glucose + phosphate-limited	2.85(R3HBA)	1.5	Guevara-Martínez et al., 2015
*E. coli* (phaA-HBD-BCH)	Glucose	10.3 (S3HBA)	0.12	Lee et al., 2008
*Clostridium coskatii* [p83_tcb]	Syngas, anaerobic culture	0.1 (R3HBA)	1.4·10^−4^	Flüchter et al., 2019
*Clostridium coskatii* [p83_tcb](thlA-ctfA/ctfB-bdhA)	Fructose, anaerobic culture	2.25 (R3HBA)	0.032	Flüchter et al., 2019
*S. cerevisiae* (ERG10- hbd-tesB)	Ethanol biotransformation under aerobic conditions	12 (S3HBA)	0.06	Yun et al., 2015
*Arxula adeninivorans* (thl-phaB)	Glucose fed-batch followed by ethanol feeding	3.78 (R3HBA)	0.043	Biernacki et al., 2017
*E. coli* BL21 (t3-rx- zwf)	Nitrogen limited fed-batch cultivation with glucose as substrate	16.3 (R3HBA)	1.52	Perez-Zabaleta et al., 2019
*Synechocystis* sp. (phaA-phaB1-TesB)	Photosynthetic cultivation	1.84 (R3HBA)	7.7·10^−3^	Wang et al., 2018
*E. coli* AF1000 (t3-rx)	Glucose, xylose and arabinose as substrate	0.54 (R3HBA)	0.029	Jarmander et al., 2015
*E. coli* (phaA -bktB-phaB-hdb-tesB-ΔsdhAΔiclR)	Glycerol as carbon and energy source, aerobic	2.0 (R3HBA)	0.042	Miscevic et al., 2019
		3.71 (R3HVA)	0.077	

a *R3HBA, (R)-3-hydroxybutyric acid; R3HDD, (R)-3-hydroxydodecanoic acid; S3HBA, (S)-3-hydroxybutyric acid; S3HVA, (S)-3-hydroxyvaleric acid*.

In *E. coli*, acetate is often produced concomitantly with R3HBA (Gao et al., 2002; Tseng et al., 2009; Guevara-Martínez et al., 2015), decreasing product yield and lowering the growth rate at concentrations as low as 0.5 g L^−1^; thus, difficulting the use of high-cell-density cultures with high volumetric productivities (Nakano et al., 1997; Roe et al., 2002). Perez-Zabaleta et al. (2019) studied the production of R3HBA using the native *E. coli* acyl-CoA thioesterases (*fadM, tesA, tesB, ybgC, ydiI*, and *yciA*). These enzymes are also active with acetyl-CoA as substrate (McMahon and Prather, 2014), and therefore could contribute to the production of acetate. In this work, the impact of deletion of genes involved in the production of acetic acid (*poxB, pta*, or *iclR*) on R3HBA producing fed-batch cultures was investigated using *E. coli* AF1000. Also, several *E. coli* strains were compared including *E. coli* AF1000 and BL21 as low acetate-forming R3HBA production platforms. All the strains carrying β-ketothiolase (*t3*) and acetoacetyl-CoA reductase (*rx*) from *Halomonas boliviensis* and overexpressing the glucose-6-phosphate dehydrogenase gene (*zwf* ). No important reduction on acetic acid titers was found for the deletion of the aforementioned genes. The best results were obtained using strain BL21 achieving the highest R3HBA titer and volumetric productivity reported up to date (see Table 2). Interestingly, Guevara-Martínez et al. (2019) working with the same recombinant strain (*E. coli* AF1000-*t3*-*rx*-*zwf* ) showed that the deletion of *tesA* or *fadM* resulted in minor decreases in R3HBA production, while deletion of *tesB* and *yciA* decreased the R3HBA titer by 11 and 33%, respectively. These results suggest that YciA, and not TesB, is the acyl-CoA thioesterase largely responsible for R3HBA production from (*R*)-3-hydroxybutyryl-CoA in *E. coli*.

The production of S3HBA from glucose in an engineered *E. coli* strain was reported by Lee et al. (2008). The genes coding for PhaA from *R. eutropha*, (*S*)-3-hydroxybutyryl-CoA dehydrogenase (HBD) from *Clostridium acetobutylicum* ATCC824, and 3-hydroxyisobutyryl-CoA hydrolase (BCH) from *Bacillus cereus* ATCC14579 were inserted in *E. coli* BL21. Under fed-batch cultivation with glucose as substrate, 10.3 g L^−1^ of S3HBA and 65 g L^−1^ of biomass were accumulated in 38 h. The yield of S3HBA production was low compared with that of R3HBA from glucose found by Gao et al. (2002). Lee et al. (2008) interpreted this low yield as a consequence of S3HBA-CoA being an intermediate of fatty-acid β-oxidation pathway; therefore, it can be degraded into acetyl-CoA. Similarly, Tseng et al. reported the production of S3HBA and R3HBA using an *E. coli* strain harboring plasmids containing genes for different thiolases (*phaA* and *bktB* from *R. eutropha* and *thl* from *C. acetobutylicum* ATCC 824), *hbd* and *phaB* genes for the production of (*R*) and (*S*) hydroxybutyryl*-*CoA and either *tesB* or *ptb-buk* genes to remove the CoA moiety. The impact of the thiolase in the titers of the hydroxy acids was found to be low. On the other the selection of the enzyme hydrolyzing the CoA was critical. In fact, when Ptb-Buk was used no S3HBA was detected. If TesB was included, then both R3HBA and S3HBA were produced.

Recently, (*R*)-3-hydroxyvalerate (R3HVA) and R3HBA were produced in a recombinant strain of *E. coli* capable of glycerol conversion (Miscevic et al., 2019). The engineered strain (P3HA31) harbors the *phaA* and *bktB* genes (from *Cupriavidus necator* ATCC 43291) to catalyze the condensation of two acetyl-CoA molecules or an acetyl-CoA and a propionyl-CoA molecule for the formation of acetoacetyl-CoA and 3-ketovaleryl-CoA, respectively. Two genes for the reduction of acetoacetyl-CoA were inserted, *phaB* from *C. necator* and *hdb* from *Clostridium acetobutylicum* ATCC 824 catalyzing the formation of (*R*)-3-hydroxybutyril-CoA and its *S* stereoisomer, respectively. Finally, *tesB* was introduced for the production of the corresponding hydroxycarboxylic acids. The strain was further optimized to increase the propionyl-CoA pool from succinyl-CoA by deregulating the glyoxylate shunt (mutating *iclR*) and by inactivating the oxidative TCA cycle gene *sdhA*, thus blocking the conversion of succinate to fumarate. The double mutant P3HA31Δ*sdhA*Δ*iclR* achieved a concentration of 3.71 g L^−1^ of R3HVA, 2.97 g L^−1^ of propionate and nearly 2 g L^−1^ of R3HBA. Despite the fact that *hdb* was expressed, results suggest that PhaB was catalytically more active than Hbd since higher titers in (*R*)-3-HB/HV than (*S*)-3-HB/HV were found. Alternatively, this might be the consequence of a larger abundance of NAPDH compared to NADH, as PhaB and Hbd are NADPH-dependent and NADH-dependent, respectively. Tseng et al. (2010) previously reported that a similar *E. coli* engineered strain was able to produce R3HBAs and (*R*) and (*S*) 3HVAs. When glycerol was used as carbon source, 0.60 g L^−1^ R3HVA, 0.19 g L^−1^ S3HVA, 0.58 g L^−1^ R3HBA, and nearly 1.2 g L^−1^ acetate were produced.

Finally, a third alternative for the hydrolysis of CoA from (*R*)-3-hydroxybutyryl-CoA, besides TesB and Ptb-Buk, was designed based on the use of a propionyl CoA transferase (Pct). Pct works by transferring a CoA from (*R*)-3-hydroxybutyryl-CoA to another short-chain fatty acid. Matsumoto et al. (2013) engineered *E. coli* BW25113 by inserting a plasmid containing the *phaA* and *phaB* genes from *R. eutropha* and *pct* from *Clostridium propionicum*. This *pct* gene was selected since catalyzes the transfer reaction of CoA between R3HBA and acetate (Jossek et al., 1998). Cells were cultured for 24 h in test tubes at 30°C with 10 g L^−1^ glucose and acetate concentrations in the 0–10 g L^−1^ range. Acetic acid enhances R3HBA production when Pct is expressed as will act as the molecule receiving the CoA moiety and will serve as substrate for the condensation reaction of two acetyl-CoAs catalyzed by PhaA. Results showed that the maximum concentration (9.0 g L^−1^) and volumetric productivity (0.22 g L^−1^ h^−1^) were attained at a concentration of 6.6 g L^−1^ of acetic acid. Unfortunately, no efforts were made to investigate the performance of the engineered strain using a fed-batch culture where acetic acid and glucose concentrations could be controlled independently, possibly increasing product titer and volumetric productivity.

## Alternative Substrates and Microorganisms for 3HA Production

Decreasing the cost of industrial PHA production requires the use of abundant and low-cost carbon and energy substrates such as molasses, glucose, sucrose derived from sugar cane, sugars produced from lignocellulose, one-carbon compounds (formate and methanol) and gaseous substrates (syngas, knallgas, and methane). This is also true for the production of the monomers constituting PHAs.

Regarding the use of lignocellulosic hydrolyzates, Wang and Liu (2014) discovered that in batch cultures of *B. cepacia*, the concentration of R3HBA released during growth was stimulated by nitrate and chloride ions. The concentration of R3HBA reached 16.2 g L^−1^ in hydrolysates of *Paulownia elongate*, however, this concentration could not be achieved in model hydrolyzates. The production of 3HBA with *Halomonas* sp. KM-1 has also been shown with sugars obtained from lignocellulosic materials. Using saccharified Japanese cedar as carbon source, a concentration of 21.1 g L^−1^ R3HBA was obtained with a yield of 89% (based on initial PHB) after shifting from an aerobic accumulation phase to a microaerobic PHB depolymerization phase (Kawata et al., 2015). Interestingly in this microorganism, the R3HBA titer obtained without the addition of urea at the onset of the microaerobic phase was lower compared to adding 7.1 g L^−1^ of urea. Unfortunately, it is not clear whether the effect of urea is due to an increase in media pH or to its consumption.

Jarmander et al. (2015) reported the construction of an *E. coli* strain capable of producing R3HBA from xylose, glucose and arabinose harboring the acetoacetyl-CoA thiolase (*t3*) and acetoacetyl-CoA reductase (*rx*) genes from *H. boliviensis*. The conversion of 3-hydroxybutyryl-CoA to R3HBA was assumed to be catalyzed by the *E. coli* native TesB enzyme. Albeit this work represents an interesting achievement from a genetic manipulation perspective targeting the production of new compounds from lignocellulosic derived sugars, the highest R3HBA yield on mixed sugars was 0.23 g/g with a 3HBA titer of 0.54 g L^−1^ under nitrogen depleted conditions in batch culture. On the other hand, the highest concentration of R3HBA (1.87 g L^−1^) was achieved in a nitrogen fed-batch culture with an overall yield of 0.05 g R3HBA g sugars^−1^.

Although the accumulation of PHB from knallgas is well-documented (see section Substrates for the Production of Polyhydroxyalkanoates and Its Monomers), no evidence exists in the open literature regarding the production of 3HBA or 3HAs from knallgas, despite *A. lata* being one of the first bacteria where R3HBA production was demonstrated (Lee et al., 1999). Similarly, the accumulation of PHB in methanotrophs is well documented (for a recent review see Strong et al., 2016), however, no known organism has been reported to produce 3HBA or any other 3HA either from *in-vivo* depolymerization of the accumulated PHB, through an engineered pathway or from the *ex-vivo* depolymerization of the extracted PHB.

A notable exception regarding the use of C1 compounds for the production of 3HAs is the use of methanol for the obtention of R3HBA. *Methylobacterium rhodesianum* MB 126 was genetically modified by knocking-out the 3-hydroxybutyrate dehydrogenase gene. Since the mutant strain still exhibits growth in R3HBA, transposon mutagenesis was used to obtain a double mutant unable to grow on R3HBA. The double mutant was shown to have, along with a lack of 3-hydroxybutyrate dehydrogenase, an incomplete citric acid cycle due to a defective lipoic acid synthase (LipA). In fed-batch culture, using methanol as the sole carbon and energy substrate, 2.8 g L^−1^ of R3HBA were obtained after accumulating PHB under nitrogen limitation and inducing its degradation under a carbon limited and nitrogen-rich condition (Hölscher et al., 2010).

All the aforementioned genetic modifications lead to the production of 3HBA or 3HAs under aerobic conditions. Flüchter et al. (2019) constructed a genetically modified strain of *Clostridium coskatii* harboring the thiolase A gene (*thlA*; CA_C2873) and the acetoacetyl-CoA:acetate/butyrate CoA transferase genes (*ctfA/ctfB*; CA_P0163/ CA_P0164) from *C. acetobutylicum* ATCC 824 and the 3-hydroxybutyrate dehydrogenase gene (*bdhA*; CDIF630_02933) from *Clostridium difficile* DSM 27543. The engineered pathway directs acetyl-CoA to acetoacetyl-CoA using thiolase A, which is converted into acetoacetate using the acetoacetyl- CoA:acetate/butyrate CoA transferase and the final conversion of acetoacetate to 3-hydroxybutyrate by 3-hydroxybutyrate dehydrogenase. Under heterotrophic conditions with fructose as carbon and energy source and under anaerobic condition, 2.3 g L^−1^ of R3HBA accumulated in 60 h of batch culture and 3.9 g L^−1^ of acetate were concomitantly produced. Under autotrophic conditions using syngas (CO 40 mol %, H_2_ 40 mol %, CO_2_ 10 mol % and N_2_ 10 mol %) a concentration of 0.1 g L^−1^ of R3HBA was obtained along with 2.1 g L^−1^ of acetate. Although the achieved concentrations, especially under autotrophic conditions are low, this study represents an important proof of concept for the utilization of inexpensive and abundant gaseous substrates. Interestingly, although the production of acetate should be reduced, it cannot be completely eliminated as acetate is used in the reaction catalyzed by the acetoacetyl- CoA:acetate/butyrate CoA transferase to accept the CoA molecule in the step leading to the production of acetoacetate. In a previous report, Woolston et al. (2018) achieved comparable titers both under heterotrophic and autotrophic growth. However, the product obtained by Woolston et al. was (S)-3-HBA instead of R-3-HBA due to an engineered pathway using the *phaA* gene from *C. necator*, the NADH-dependent (*S*)-3-hydroxybutyryl-CoA dehydrogenase from *C. acetobutylicum* and the thioesterase *tesB* from *E. coli*.

The production of (*S*)-3-HBA has been also demonstrated in metabolically engineered *Saccharomyces cerevisiae*, Yun et al. (2015) reported one of the few modifications in yeasts aimed at the production of hydroxyacids, where the following genes were introduced: acetyl-CoA C-acetyltransferase (*erg10p* from *S. cerevisiae* BY4741) for the condensation of two molecules of acetyl-CoA into acetoacetyl-CoA, NADH-dependent acetoacetyl-CoA reductase (*ACR*, hbd from *C. acetobutylicum* ATCC 824) converting acetoacetyl-CoA to (S)-3-hydroxybutyryl-CoA and 3-hydroxybutyryl-CoA thioesterase (*tesB* from *E. coli* K-12 MG1655) to remove the CoA molecule from (*S*)-3-hydroxybutyryl-CoA. Using ethanol as the substrate in fed-batch cultivation, 12 g L^−1^ of (*S*)-3-HBA were accumulated in 200 h. Small amounts of glycerol were accumulated during the first half of the culture (close to 6 g L^−1^) but they were reduced to zero by the end of the culture. A similar strategy was used by Biernacki et al. (2017) for the production of R3HBA in *Arxula adeninivorans* after inserting the *thl* gene from *C. acetobutylicum* ATCC 824 and *phbB* from *C. necator* H16. In fed-batch cultures with glucose as the carbon source, ethanol is produced under hypoxic conditions during the first 50 h of culture. A shift to aerobic conditions promotes ethanol assimilation and R3HBA production reaching a maximum of 3.78 g L^−1^.

Finally, the use of photosynthetic organisms for 3HA production has also been explored. Wang et al. (2018) modified the cyanobacteria *Synechocystis* sp. After inserting the genes *phaA, phaB1*, and *tesB* a concentration of 1.84 g L^−1^ of R3HBA was obtained at the end of 10 days of photoautotrophic cultivation.

Most of the available literature on the direct microbial production of HAs (without accumulation of PHAs) deals with the obtention of short-chain length hydroxy acids. Notable exceptions are the engineered systems based on *Pseudomonas putida* strains. Chung et al. (2009) constructed a novel pathway in *Pseudomonas putida* KTOY01, a mutant of *P. putida* KT2440 unable to accumulate PHA due to a PHA synthesis operon knockout, by expressing the *tesB* gene and knocking the genes *fadB* and *fadA* to create a β-oxidation-pathway-deficient mutant. This strain accumulated 7.27 g L^−1^ of R3HAs, with over 96% mol of (*R*)-3-hydroxydodecanoic acid when lauric acid was added into the culture broth. However, lauric acid is a related carbon source making this process closer to biotransformation rather than synthesis. Similar work was performed by Chung et al. using *Pseudomonas entomophila* as the host strain. When tetranoic acid and dodecanoic acid were used as related carbon sources in shake flask cultures, 6.65 g L^−1^ 3-hydroxytetradecanoic acid and 4.6 g L^−1^ 3-hydroxydodecanoic acid were obtained. No reports of the production of mcl-HAs are available in the literature, however, the work of Agnew et al. (2012) showing the production of C_12_ and C_14_ polyhydroxyalkanoates from glucose in *E. coli* could be a starting point for the introduction of a thioesterase such as TesB.

## Secretion of PHAs

A possible approach to decrease the cost of PHAs and 3HAs production is to devise simplified methods for the recovery of the accumulated polymers. Although the production of 3HAs from recovered PHAs requires more steps than the direct fermentation strategies presented in the previous section, starting from pure or partially purified PHAs could lead to a simplified downstream processing. Moreover, the production 3HAs esters, as outlined in section Chemical and Enzymatic Hydrolysis of Recovered PHAs starts from PHAs, and thus it can benefit from simplified methods for the recovery of these polymers. A recurrent strategy is the induction of cell lysis to release the PHA granules.

Resch et al. (1998) expressed the *Alcaligenes eutrophus phbCAB* genes and the cloned lysis gene E of bacteriophage PhiX174 in *E.coli* cells, which allowed to perform PHB synthesis and at the same time generate the E-lysis tunnel structure that is characterized by a small opening with edges in the transmembrane envelope (Resch et al., 1998). E-lysis produces holes approximately of equal size to the diameter of the cells (producing lysis) and releases around 90% of the PHB. The PHB granules vary in size and have the tendency of self-aggregation. Based on these facts and the structural analysis of protein E it was postulated as a way to obtain free PHB granules in the extracellular medium. Similarly, a *P. putida* KT2440 mutant was constructed by expressing two proteins from the pneumococcal bacteriophage EJ-1, an endolysin (Ejl) and a holin (Ejh) and the mutation of the *tolB* gene to reduce the integrity of the membrane and promote a lysis sensitivity. After inducing the accumulation of PHAs and the lysis of cells, 0.28 g L^−1^ of PHAs were recovered using direct extraction of the wet biomass with ethyl acetate (Martínez et al., 2011). Using the endolysin (Ejl) and a holin (Ejh) system, but under the control of a promoter induced by xylose but inhibited by glucose, nearly two-thirds of the PHB accumulated in *Bacillus megaterium* using glucose as carbon source was released into the broth after 20 h of culture post glucose exhaustion. A fraction of the PHB remains associated with the cell debris, possible trough hydrophobic interactions (Hori et al., 2002).

A different but related approach was presented by Borrero-de Acuña et al. (2017). A programmable lysis system was built based on the expression of lysozyme and tested in *Pseudomonas putida* KT2440 under conditions promoting growth or PHA accumulation. The lytic system did not affect the biomass yield or growth rate of *P. putida* under balanced growth but did reduce the biomass production by 25% when conditions permissive of PHA accumulation were applied. Notwithstanding, the PHA content was kept and after the induction of the lytic system, nearly 75% of the accumulated polymer was recovered. The released polymer was recovered by mixing the fermentation broth with chloroform at a 18:1 (v/v) ratio followed by phase separation, representing an excellent reduction in solvent and energy use.

Another strategy for the recovery of PHAs is the use of external cell lytic agents such as the *Bdellovibrio bacteriovorus* HD100 bacterium, an obligate predator of other gram-negative bacteria which acts as a lithic agent for the recovery of intracellular bioproducts of industrial interest (Martínez et al., 2016). *B. bacteriovorus* HD100 was genetically modified by eliminating the PHA depolymerase gene in order to prevent the breakdown of the recovered PHAs. After allowing the accumulation of PHAs in *P. putida, C. necator*, and a recombinant *E. coli* strain, each culture was infected with a suspension of *B. bacteriovorus* cells. Sixty-five percentage of the PHA accumulated in *P. putida* could be recovered at high cell densities using this method. The polymer was directly extractable from the wet biomass of the co-cultures, thus avoiding the need for biomass drying. When *C. necator* cells were used as prey in low concentration (PHB titer less than 1 g L^−1^), the PHB recovery yield was 80%.

Currently, efforts are focused on the creation of “leaking” bacteria for an easy “export” of PHB, this is the implementation of secretion mechanisms in which a cell breakdown is not required in a chemical or mechanical way and the biomass could be recycled to implement a semi-continuous PHB production system. Protein secretion is the main route by which bacteria not only interact with the extracellular environment, but also secrete products that are essential for cells, which include adhesion, pathogenicity, adaptation, and in some cases enzymatic degradation. Hence, the gram-negative bacteria have developed a wide variety of pathways for the secretion of different products into the extracellular matrix while maintaining the integrity of the cellular structure (Henderson and He, 2009; Costa et al., 2015).

Type I secretion systems (T1SS) or ABC transporters (ATP-binding cassette) are heterotrimeric complexes that are composed of three segments that interact with each other (Nicaud et al., 1986). Type II secretion systems (T2SS) are one of the best-known secretion systems because they are conserved in most gram-negative bacteria. Type II systems are able to transport folded proteins from the periplasm to the extracellular environment (Green and Mecsas, 2016). T1SS and T2SS are the most used secretion systems in biological engineering and therefore the most studied in detail. These two systems stand out for the fact that each one of them recognizes a sequence through its peptide ends as an objective, that is, the signalized sequence can be fused with other proteins which causes the cell to target the new fusion protein for their respective secretion. Fusion proteins have been applied to indirectly secrete PHB molecules, this has been reported by Linton et al. (2012), who initially conducted a study to evaluate the efficacy and viability of the secretion systems used by *E. coli*. The signal peptides corresponded to HlyA (T1SS), TorA (T2SS, TAT), GeneIII (T2SS, Sec), and PelB (T2SS, Sec) (Linton et al., 2012). The results obtained show that the PelB system was not effective in the translocation of GFP, while the other two T2SSs successfully exported GFP to the periplasm and with respect to the HlyA system, also secrete the GFP protein to the extracellular medium (Linton et al., 2012). Based on these results, the HlyA signal peptide fusion protein and a fascine that associates with the PHB granules were used to bind the signaling sequence to the PHB granule, leading to the secretion of PHB to the extracellular medium (Rahman et al., 2013). Results indicate that after 48 h of culture 36% of the total PHB produced by the secretory strain was collected in the secreted fraction while the remaining 64% corresponded to the internal fraction. SEM images of the PHB accumulating *E. coli* strain with and without the secretory system show that PHB is not excreted as granules, but as an amorphous material. This evidence, combined with the low secretion found, suggest that the system cannot export the intact PHB granules. Sabirova et al. (2006) constructed an *Alcanivorax borkumensis* SK2 mutant capable of PHA hyperproduction. Interestingly, this mutant release part of the produced PHA to the extracellular medium when it was cultured in alkanes without cell lysis. The secretion mechanism remains unknown.

## Conclusion and Perspectives

As outlined in this review, after 20 years of the discovery of the *in-vivo* hydrolysis of PHB accumulated in *Azohydromonas lata*, and even though this system remains as the one showing the highest titer of (*R*)-3-hydroxybutyrate, a large body of knowledge have been accumulated regarding the production of other, potentially more industrially useful, 3-hydroxyalkanoic acids from diverse substrates including fatty acids and sugars derived from lignocellulosic materials. In this regard, the production of 3-hydroxyacids directly from its precursors, and without the accumulation of PHAs, in recombinant strains show the potential of transferring this production system into a wide range of hosts to expand the number of substrates that can be used for the production of these valuable compounds.

Thanks to the availability of techniques for genetic manipulation, *E. coli* served as the preferred host for the introduction of new pathways for the direct production of 3HAs. Since the first reports dealing with the production of R3HBA using the native *E. coli* thioesterases, significant efforts were performed to increase titer, yield, and productivities and to expand the range of 3HAs that can be obtained in *E. coli*.

However, more research is needed for bacterial production of these hydroxy acids to reach a level of industrial use. Research needs include increasing titer and productivities in *E. coli* from sugars and other readily available substrates, as well as achieving its production from inexpensive gaseous substrates such as methane, syngas, and knallgas. Up to date, only the production of 3-hydroxybutyrate has been demonstrated from syngas, remaining the production of this acid from knallgas and methane, as well as the production of mcl-hydroxy acids a completely unexplored area of research.

Finally, the secretion and lytic systems, mainly developed during the last two decades, open the possibility of a facile and inexpensive PHA recovery, combined with efficient chemical or enzymatic hydrolysis methods, they can constitute a powerful combination for gaining access to a wide range of 3-hydroxy acids.

## Author Contributions

All the authors performed literature search and drafted sections of the manuscript. LY, RC, and FS drafted most of the sections dealing with PHA accumulation, 3HA production and alternative PHA recovery strategies. AV-F drafted sections dealing with the different substrates used for PHA production and created Figure 1. All authors revised the manuscript and approved the final version.

### Conflict of Interest

The authors declare that the research was conducted in the absence of any commercial or financial relationships that could be construed as a potential conflict of interest.
